# Outer Membrane Vesicles Formed by Clinical *Proteus mirabilis* Strains May Be Incorporated into the Outer Membrane of Other *P. mirabilis* Cells and Demonstrate Lytic Properties

**DOI:** 10.3390/molecules29204836

**Published:** 2024-10-12

**Authors:** Dominika Szczerbiec, Sława Glińska, Justyna Kamińska, Dominika Drzewiecka

**Affiliations:** 1 Department of Biology of Bacteria, Faculty of Biology and Environmental Protection, University of Lodz, Banacha 12/16, 90-237 Lodz, Poland; dominika.szczerbiec@biol.uni.lodz.pl (D.S.); justyna.kaminska456@gmail.com (J.K.); 2 Laboratory of Microscopic Imaging and Specialized Biological Techniques, Faculty of Biology and Environmental Protection, University of Lodz, Banacha 12/16, 90-237 Lodz, Poland; slawa.glinska@biol.uni.lodz.pl

**Keywords:** OMVs, blebs, fusion, killing, bactericidal effect, O78 serogroup, O77 antigen, ELISA

## Abstract

Outer membrane vesicles (OMVs) are extracellular structures, ranging in size from 10 to 300 nm, produced by Gram-negative bacteria. They can be incorporated into the outer membrane of a recipient’s cells, which may enable the transfer of substances with lytic properties. Due to the scarce information regarding the OMVs produced by *Proteus mirabilis*, the aim of this study was to test the blebbing abilities of the clinical *P. mirabilis* O77 and O78 strains and to determine the blebs’ interactions with bacterial cells, including their possible bactericidal activities. The production of OMVs was visualised by Transmission electron microscopy (TEM). The presence of OMVs in the obtained samples as well as the phenomenon of OMV fusion to recipient cells were confirmed by Enzyme-Linked ImmunoSorbent Assay (ELISA) and Western blotting assays. The bacteriolytic activity of the OMVs was examined against *P. mirabilis* clinical strains and reference *Staphylococcus aureus* and *Escherichia coli* strains. It was shown that each of the two tested *P. mirabilis* strains could produce OMVs which were able to fuse into the cells of the other strain. The lytic properties of the O78 OMVs against another *P. mirabilis* O78 strain were also demonstrated. This promising result may help in the future to better understand the mechanisms of the pathogenesis and to treat the infections caused by *P. mirabilis*.

## 1. Introduction

*Proteus mirabilis* are Gram-negative bacteria, living in soil, in the organisms of wild and domestic animals and human beings. The bacilli may play the role of commensals or even as symbionts of higher organisms. However, they are also considered to be human and animal facultative pathogens, as, in some circumstances, *P. mirabilis* may cause severe infections [1]. In particular, the bacteria pose a threat of nosocomially acquired urinary tract infections (UTIs) complicated by urinary stone formation. The infections are frequently connected with the use of urinary catheters in hospitals. *P. mirabilis* bacilli may produce a large spectrum of pathogenic factors, enabling them to gradually reach the urinary tract niches in the ascending UTI. Among these factors, the best-recognised ones have been flagella and the swimming and swarming capacities, adhesin and biofilm formation, urease and urinary stone formation, haemolysins and toxicity, protease and host protein degradation, as well as lipopolysaccharide (LPS) with its endotoxic and antigenic properties [2]. Outer membrane vesicles (OMVs) are one of the less known *P. mirabilis* virulence factors. These 10–300 nm diameter structures are formed by blebbing from the outer membrane in possibly all Gram-negative bacteria, including the genus *Proteus*. Besides the outer membrane compounds, such as lipopolysaccharides, phospholipids, and proteins, OMVs (blebs) may contain some substances coming from the periplasm or the cytoplasm, and, in some cases, the second membrane formed by the inner (cytoplasmic) membrane is also present inside [3].

OMVs are an important virulence factor of various microorganisms. In the lumen of these structures, there have been detected compounds produced or removed by the host bacteria. The blebs may play the role of carriers of many substances to other bacteria living in the biofilm structure or free (e.g., DNA, RNA, autolysins, and quorum-sensing molecules), or to the eukaryotic host tissues (e.g., toxins). The transport of some substances from the producer to the other cells involves the mechanisms enabling the incorporation of the blebs to the recipient cells. Indeed, the vesicle may merge with the outer membrane of the recipient cell, delivering its cargo to the cell’s periplasmic space [4,5].

However, there are only a few papers available on the OMVs produced by the bacteria from the genus *Proteus*. *Proteus* OMVs have been reported as possessing antagonistic properties against eukaryotic or bacterial cells and participating in the pathogenesis of these rods, trapping such virulence factors as phospholipase C, proelastase, proteases, and heamolysins [6]. Gao et al. [7] found that *P. mirabilis* OMVs present in boar semen had a toxic and lethal effect on the spermatozoids caused by the presence of LPSs in the blebs, thus decreasing the semen quality during storage. Wang et al. [8] investigated the potential role of *P. mirabilis* OMVs in bone tissue diseases and, surprisingly, demonstrated their osteoprotective properties.

Li et al. [9] reported the lytic effect of *P. vulgaris* OMVs on the cells of other Gram-negative as well as Gram-positive bacterial species. Lysis was stimulated by peptidoglycan hydrolases (autolysins) which were closed in the lumen of the blebs. Various mechanisms of the blebs’ lytic action on Gram-negative and Gram-positive bacterial cells have been studied and explained [10]. In Gram-positive cells, lysis occurs in the place of contact between the vesicle and the cell wall. The lysis of Gram-negative cells involves the incorporation of the vesicle membrane into the cell wall outer membrane and the release of autolysins into the periplasmic space. Thus, OMVs may be used as antibiotic substances. There are also other potential applications for these structures in practice, including vaccine development [11].

In this work, we have studied the efficiency of blebbing and the properties of the OMVs released by clinical *P. mirabilis* strains, representing O77 and O78 serogroups, which are widespread among patients in central Poland [12].

## 2. Results

### 2.1. Determination of the Late Logarithmic Growth Phase in P. mirabilis Cultures

**Figure 1:**
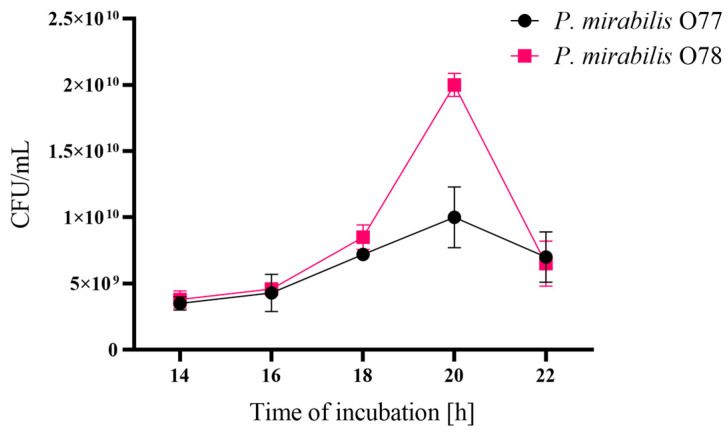
The growth curves of the *P. mirabilis* O77 and O78 strains, presented as colony-forming units (CFUs)/mL—mean ± standard deviation (SD) during the incubation time.

The logarithmic growth phase was determined for the *P. mirabilis* O77 and O78 strains. This growth phase is characterised by the most intense cell division and, consequently, according to the literature data, the highest number of OMVs is produced during this period [13]. For both *P. mirabilis* O77 and O78 strains cultivated on nutrient broth medium, the peak of the CFUs was visible in the 20th hour of incubation (Table S1, Supplementary Materials). The results were also confirmed on Luria broth (LB) medium. As seen in Figure 1, the end of the logarithmic growth phase of both strain cultures could be estimated at the 19th hour of cultivation. After 20 h of incubation, a decrease in the number of bacterial cells and their death could be observed. Therefore, the 19th hour of the *P. mirabilis* strain cultivation was considered as the most optimal time for the production of OMVs.

### 2.2. P. mirabilis Strains Are Able to Produce OMVs

Five OMV samples were studied during the course of the research. The samples OMV 3 and 8–9 were obtained from the O77 strain, while the samples OMV 7 and 10 came from the O78 strain (Table 1).

**Figure 2:**
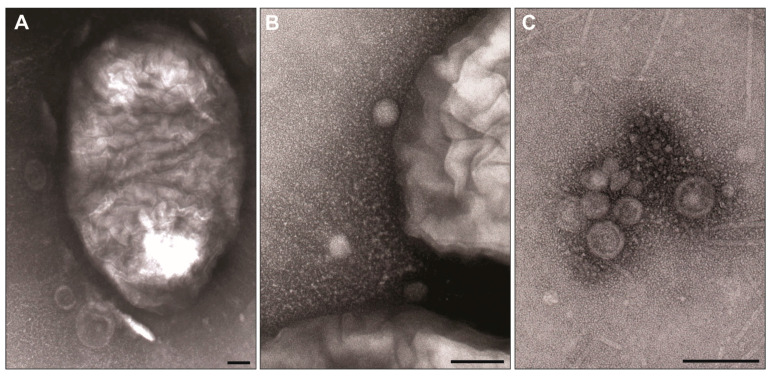
Transmission electron microscopy (TEM) of the OMVs from *P. mirabilis*. Micrographs showing the OMVs released from the *P. mirabilis* O77 (**A**) and *P. mirabilis* O78 (**B**) cells, and the OMV sample from *P. mirabilis* O78 (OMV10) (**C**). Bars, 100 nm.

The blebbing processes were visualised for the *P. mirabilis* O77 and O78 cells using Transmission electron microscopy (TEM) (Figure 2A,B). The presence of the vesicles was also confirmed in the obtained OMV samples (Figure 2C). The observed spherical structures, surrounded by a clearly visible outer membrane, varied in size. The analysis of the TEM micrographs showed that the diameter of the dried bacterial cells ranged from 515 to 837 nm and their length ranged from 1025 to 1736 nm, while the diameter of the O77 (OMV3) and O78 (OMV10) blebs ranged from 30 to 200 nm. A similar size range was confirmed using the Zetasizer Ultra Red. The average size of the bacterial cells was estimated to reach 2381 nm (SD ± 165.7) in the *P. mirabilis* O77 strain and 2269 nm (SD ± 625.8) in the *P. mirabilis* O78 strain. The mean diameter of the O77 OMVs in the OMV3 sample was 118.3 nm. The following two main groups of these structures were detected: 86.48% were vesicles as big as 143 nm in diameter, while 13.52% were small vesicles with a diameter close to 45 nm. The mean zeta potential of the O77 OMVs was weakly negative (−8.224 mV, SD ± 1.423), varying from −9.761 mV to −6.953 mV.

**Figure 3:**
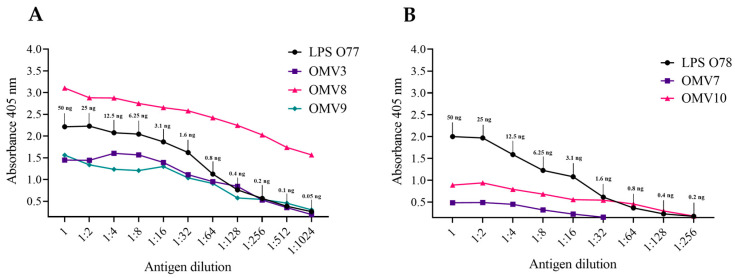
Reactions (mean) in the Enzyme-Linked ImmunoSorbent Assay (ELISA) of the OMV samples obtained from the *P. mirabilis* O77 cultures with the O77 antiserum (**A**) and from the *P. mirabilis* O78 cultures with the O78 antiserum (**B**), and the respective *P. mirabilis* LPSs—controls.

To assess the OMV production, the LPSs building the OMVs were identified in the Enzyme-Linked ImmunoSorbent Assay (ELISA) and compared to the control LPSs extracted from the biomass of the respective producing strains (Figure 3, Table S2—Supplementary Materials). Additionally, this assay allowed us to estimate the amount of LPSs in the obtained OMV samples. As various samples might be coated on the ELISA wells with different effectiveness, we took the titres into account. Both of the tested *P. mirabilis* strains produced vesicles but with different intensities. It can be estimated that, in the titre (dilution of 1:1024) of the control sample (0.05 ng of O77 LPSs), the absorbance of OMV8 was six times higher. So, the LPSs in the OMV8 sample reached the amount of 6 × 0.05 ng = 0.3 ng in a 1:1024 dilution. Therefore, it was assumed that, in the undiluted OMV8 sample, its LPS amount reached about 300 ng/50 µL. Similarly, it was estimated that there was about 13 ng/50 µL of LPSs in the OMV7 sample. As the absorbance in its titre (dilution at a ratio of 1:32) was four times lower than in the control (1.6 ng of O78 LPSs), the amount of OMV7 LPSs in this dilution should be 1.6 ng/4 = 0.4 ng; thus, in the undiluted sample, it was 32 times higher. The OMV3, OMV9, and OMV10 samples contained about 50 ng/50 µL of LPSs (the same titres as in the respective LPS controls).

Based on the above results, the efficiency of the OMV production by the studied *P. mirabilis* strains was calculated. As shown in Table 1, it differed between the samples coming from different cultures and strains. The most abundant sample was OMV8 obtained from *P. mirabilis* O77.

### 2.3. OMVs May Be Incorporated into Recipient Bacterial Cells

**Figure 4:**
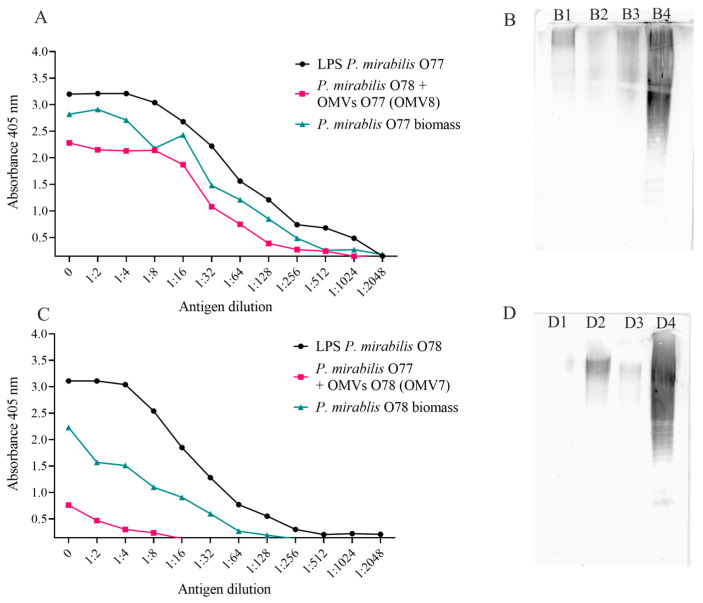
ELISA with the O77 antiserum and the tested samples or control (**A**); Western blot with the O77 antiserum (**B**), where (**B1**)—*P. mirabilis* O77 cells + OMV7 (O78), (**B2**)—*P. mirabilis* O78 cells + OMV8 (O77), (**B3**)—OMV8 (O77), and (**B4**)—O77 LPSs (a positive control). ELISA with the O78 antiserum and the tested samples or control (**C**); Western blot with the O78 antiserum (**D**), where (**D1**)—*P. mirabilis* O77 cells + OMV7 (O78), (**D2**)—*P. mirabilis* O78 cells + OMV8 (O77), (**D3**)—OMV7 (O78), and (**D4**)—O78 LPSs (a positive control).

In order to examine if the membrane vesicles obtained from the *P. mirabilis* O77 and O78 strains might cross-integrate into the cells of the two strains, a fusion test was performed. Using ELISA and Western blotting techniques, O77/O78 antigens were detected, respectively, in the cells of the bacteria, which were embedded in the outer membrane. As shown in Figure 4A, the O77 antiserum strongly reacted with the O77 LPSs (positive control) and the O77 cell biomass, as well as with the *P. mirabilis* O78 cell biomass with the integrated OMVs obtained from the *P. mirabilis* O77 strain. The last result indicated that the OMVs obtained from the O77 strain had been incorporated into the outer membrane of the *P. mirabilis* O78 strain. The above results were confirmed by Western blotting (Figure 4B). A positive reaction (the recognition of the O antigen bands) of the *P. mirabilis* O77 antiserum can be observed with the O77 LPSs (positive control, Figure 4(B4)), the OMV8 sample (OMVs produced by the *P. mirabilis* O77 strain, Figure 4(B3)), the *P. mirabilis* O77 cells with the OMV7 (O78) sample (Figure 4(B1)), and the *P. mirabilis* O78 cells with the OMV8 (O77, Figure 4(B2)) sample. The B2 bands, recognised by the O77-specific antibodies, proved the presence of the O77 antigen in the O78 strain biomass, which indicated the phenomenon of the O77 OMV fusion into the O78 cells (Figure 4(B2)) and confirmed the results obtained in the ELISA reactions. Similar results were achieved for the sample with the *P. mirabilis* O77 cells with the incorporated OMV7 (O78) sample in the reaction with the *P. mirabilis* O78 antiserum. The fusion was confirmed by the ELISA (Figure 4C) and Western blotting (Figure 4(D1)) assays; nevertheless, the reactions were much less intense.

**Figure 5:**
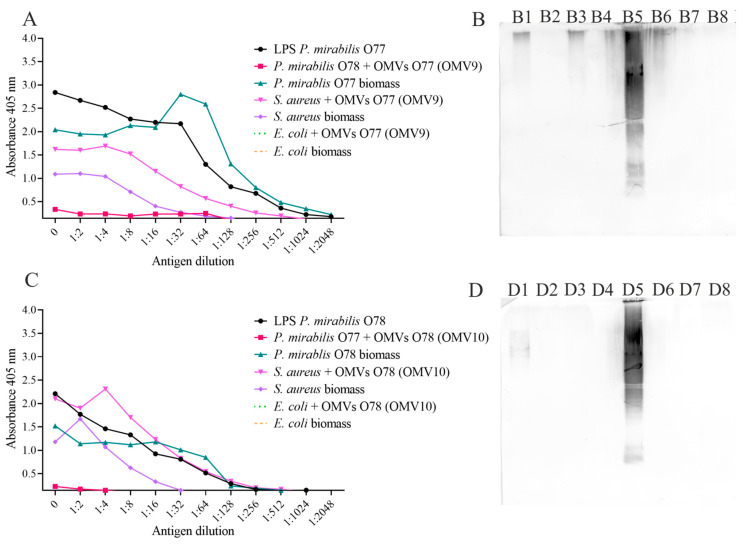
ELISA with the O77 antiserum and the tested samples (**A**); Western blot with the O77 antiserum (**B**), where (**B1**)—*P. mirabilis* O77 cells, (**B2**)—*P. mirabilis* O78 cells + OMV9 (O77), (**B3**)—OMV9 (O77), (**B4**)—*P. mirabilis* O78 cells, (**B5**)—O77 LPSs (a positive control), (**B6**)—*S. aureus* cells + OMV9 (O77), (**B7**)—*S. aureus* cells, and (**B8**)—*E. coli* cells + OMV9 (O77). ELISA with the O78 antiserum and the tested samples (**C**); Western blot with the O78 antiserum (**D**), where (**D1**)—*P. mirabilis* O78 cells, (**D2**)—*P. mirabilis* O77 cells + OMV10 (O78), (**D3**)—OMV10 (O78), (**D4**)—*P. mirabilis* O77 cells, (**D5**)—O78 LPSs (a positive control), (**D6**)—*S. aureus* cells + OMV10 (O78), (**D7**)—*S. aureus* cells, and (**D8**)—*E. coli* cells+ OMV10 (O78).

Similarly, ELISA and Western blotting techniques were performed to examine the fusion phenomenon with the following samples: OMV9 (O77) and OMV10 (O78). As before, a stronger reaction was observed for the incorporation of the O77 OMVs into the outer membrane of the *P. mirabilis* O78 cells (Figure 5A) than for the incorporation of the O78 OMVs into the O77 cells (Figure 5C). However, these reactions were weaker (a lower absorbance) than those with the OMV8 and OMV7 samples (Figure 4), so it can be assumed that the fusion proceeded less efficiently. In the course of the experiment, two reference strains, Gram-negative *Escherichia coli* and *Staphylococcus aureus* as a representative of Gram-positive bacteria, were used in the ELISA and Western blotting tests. The OMV9 and OMV10 samples did not incorporate into the outer membrane of *E. coli* (Figure 5A,(B8),C,(D8)), while, surprisingly, *S. aureus* cells were found to cross-react with *P. mirabilis* O77 and O78 antisera in the ELISA (Figure 5A,C). However, almost no reaction was observed in the Western blotting assay with *S. aureus* biomass and *S. aureus* after fusion (Figure 5(B6,B7,D6,D7)). This confirms that the *P. mirabilis* OMVs were not integrated into the *E. coli* or *S. aureus* cells and, most probably, the ELISA reaction with the *S. aureus* biomass was non-specific.

### 2.4. OMVs Produced by the P. mirabilis O78 Strain Showed Bacteriolytic Activity

**Figure 6:**
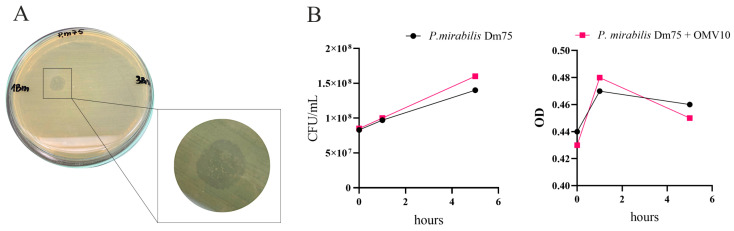
The growth inhibition zone of the *P. mirabilis* Dm75 (O78) strain by the OMV10 sample obtained from *P. mirabilis* 1Bm (O78) strain (**A**). CFUs and the optical density (OD) in the *P. mirabilis* Dm75 (O78) cell suspension—control and the cell suspension with the OMV10 sample (**B**).

In order to examine the lytic properties of the obtained OMVs, a spot assay as well as the estimation of the optical density (OD) and colony-forming units (CFUs)/mL in time were performed. In the spot test, the cytotoxic activity of the OMVs from the *P. mirabilis* O77 strain (OMV8 sample) and from the *P. mirabilis* O78 strain (OMV10 sample) against *P. mirabilis* strains (from the O77, O78, O20, and O11 serogroups), as well as *E. coli* and *S. aureus* reference strains were determined. Indeed, the lytic activity was observed in the case of the OMVs obtained from the *P. mirabilis* O78 strain (OMV10 sample), which, notwithstanding, showed these properties only against the *P. mirabilis* Dm75 strain from the same O78 serogroup (Figure 6A). The growth inhibition zones were visible in the place where the OMV droplets were added. However, the lytic activity of the OMV10 sample against the *P. mirabilis* Dm75 (O78) strain was not visible in the OD_550_ and CFUs/mL values when the OMV sample was incubated with the bacterial cell suspension (Figure 6B). The absorbance measurement in time showed almost no differences between the control and the tested sample with the OMVs. The viability of both the *P. mirabilis* Dm75 cells incubated with OMV10 and the control sample without blebs remained at a similar level throughout the experiment. After 5 h of incubation, the CFUs/mL for the sample with the OMV10 was 1.6 × 10^8^ and the OD_550_ = 0.45, and the CFUs/mL for the control sample reached 1.4 × 10^8^ and the OD_550_ = 0.46 (Figure 6B).

## 3. Discussion

The phenomenon of OMV formation by Gram-negative bacteria was first observed in 1965 in an auxotrophic *E. coli* strain [14]. Currently, the production of these structures as well as the various functions they perform are the subject of much research. Among other functions, OMVs participate in the transport of various substances such as proteins, have an impact on cellular–intercellular communication, may have immunomodulatory effects, and, importantly, antibacterial properties [15]. Although these nanostructures are produced not only by pathogenic bacteria, they are also an important pathogenicity factor. The production of OMVs as well as their characterisation and functions have been well described in many Gram-negative bacteria, especially *Pseudomonas aeruginosa* [16], *Vibrio cholerae* [17], *Salmonella typhimurium* [18], or *Neisseria meningitidis* [19].

On the other hand, according to our current knowledge, there are only two reports about the OMVs produced by *P. mirabilis* bacteria. Research conducted by Gao et al. [7] concerned the negative impact of *P. mirabilis* OMVs on boar sperm functions. It was proven that the OMVs could bind to the sperm membrane, which reduced its ability to bind to the oocyte. This study showed that membrane vesicles influenced the sperm motility and morphology, along with inducing the autophagy and apoptosis of the sperm. The apoptotic effect was connected with elevated mitochondrial membrane potential and reactive oxygen species (ROS) accumulation caused by the presence of LPSs in the blebs. However, there was no information provided on the kind of the O antigen composing the LPSs. The structure and properties of the O polysaccharide could have an important influence on the toxic effects of the *P. mirabilis* OMVs on the semen vitality. A similar mechanism of *P. mirabilis* OMV action was demonstrated by Wang et al. [8]. In these studies, the antagonistic effect on the osteoclasts was shown but, again, the vesicle O antigen was not characterised. In a mouse model, the OMVs reduced the formation of the bone cells that break down the bone tissue and, as a result, inhibited bone resorption. It is worth emphasising that a positive osteoprotective effect was observed in the case of *P. mirabilis* OMVs, contrary to the OMVs of other tested bacterial species [8]. Thus, so far, there is no comprehensive information about the OMVs produced by pathogenic *P. mirabilis* bacteria.

Therefore, the purpose of these studies was to investigate whether clinical *P. mirabilis* strains from the O serogroups widely distributed in patients might produce vesicles similarly to other Gram-negative bacteria, and to examine the capability of their fusion to recipient cells as well as their lytic properties. The *P. mirabilis* strains used in this work belong to the *Proteus* O77 and O78 serogroups, which were for the first time recognised and characterised as widespread in Lodz voivodeship, Poland. The results of this work have shown that *P. mirabilis* clinical strains isolated from human urine are able to effectively produce OMVs, which may have an impact on their pathogenicity.

To determine the presence of the examined structures in the obtained samples, we used TEM, ELISA, and Western blotting assays, methods that are characterised by high sensitivity and specificity [20]. TEM imaging allowed us to see the examined structures, displaying round morphologies and the presence of the bacterial outer membrane. Typically, OMVs have a size ranging from 10 to 300 nm [21], which was also the case for our research results on *P. mirabilis* strains. Additionally, microscopic images also showed the blebbing *P. mirabilis* O77 and O78 cells presenting a short-bacilli-like shape. The average diameter of the cells was 20 times bigger than the average vesicle diameter. Contaminants visible in the background were most likely bacterial flagella or fimbriae, which were also noticeable in the TEM images presented by other authors [22,23,24]. The slightly negative zeta potential was most probably caused by the presence of acidic groups on the membrane surface proteins, as the O77 LPS polysaccharide is neutral [25]. The zeta potential value is within the range of −30 mV to 30 mV, which may enhance the aggregative properties of the blebs [26].

According to Klimentova et al. [13], the efficiency of OMV production may be influenced by many factors such as the cultivation time, growth media, or stress. We have chosen the 19th hour of cultivation as the late logarithmic phase, when the cells are the most effective in blebbing [13]. Indeed, both strains seemed to produce large amounts of OMVs, reaching about 2000–12,000 ng of LPSs per 2 mL of sample; the blebs were only less abundant in the OMV7 (O78) sample (about 520 ng of LPSs). The efficiency of the OMV production by the tested strains was estimated based on the results obtained from the ELISA. The results showed that it may be strain-dependent, as the *P. mirabilis* O77 strain produced OMVs more actively than the O78 strain (Table 1, Figure 3). The production of OMVs by bacteria is very often increased in unfavourable conditions and depends on the pH, temperature, oxidative stress, UV radiation, osmotic pressure, and other conditions like the presence of antibacterial substances [27]. Therefore, it would be interesting to examine in the future how different conditions of cultivation and the presence of stress factors influence the efficiency of OMV production by various *P. mirabilis* strains.

Many functions performed by OMVs are related to the phenomenon of their fusion to the recipient cells, i.e., the incorporation of blebs into the outer membrane of Gram-negative bacteria. This enables gene transfer as well as the transport of many other substances, including virulence factors between bacterial cells over long distances. Simultaneously, the cargo is protected against the harmful factors of the external environment [28].

Our studies have shown that the vesicles coming from the *P. mirabilis* O77 and O78 strains are able to fuse with the non-homologous *P. mirabilis* strains. Noteworthy, the incorporations of the O77 vesicles to the O78 recipient cells were more efficient than in the opposite system, and may not only be dependent on the amount of blebs in the samples. The ELISA results proved that the OMV8 (O77) sample was the most abundant. The fusion was the most effective in the case of this sample (OMV8) and also productive in the case of the second O77 sample (OMV9), although the ELISA and Western blotting reactions of the fused OMV9 blebs were less comparable to the controls (LPSs). On the other hand, the two O78 OMV samples (OMV7 and OMV10) displayed much weaker reactions in the ELISA and Western blotting techniques after cross-fusion.

OMVs can also transport molecules with lytic activity against Gram-positive and Gram-negative bacteria, distinguishing between self-cells and nonself-cells [3]. However, it is worth emphasising that the lysis of Gram-positive and Gram-negative bacteria proceeds differently. In Gram-positive bacteria, OMVs break after contact with the cell wall and release lytic enzymes near a bacterial cell, which then digest its cell wall. In the case of Gram-negative bacteria, the vesicles are attached and incorporated into the outer membrane of the bacterial cell, thus delivering the cargo into the periplasm. As a result, the peptidoglycan is digested at multiple sites [6].

The lytic activity of OMVs has been reported for many bacteria, e.g., especially *Pseudomonas aeruginosa* [29], and also *Escherichia coli* or *Serratia marcescens*, and some other members of *Enterobacterales* [9]. However, there is only one report about OMVs produced by *P. vulgaris*, which showed antibacterial activity against both Gram-negative and Gram-positive bacteria such as *Bacillus subtilis*, *Pseudomonas aeruginosa*, *Escherichia coli*, *Mycobacterium smegmatis*, *Mycobacterium phlei*, *Lactococcus lactis*, and *Brachybacterium conglomeratum* [9]. Also, Beveridge [6] mentioned lytic *P. mirabilis* OMVs. No further and more up-to-date reports on the antagonistic properties of *Proteus* OMVs against other bacteria are available. The results of our work showed that OMVs obtained from the clinical *P. mirabilis* 1B-m (O78) strain expressed a strong cytotoxic effect on another clinical strain, *P. mirabilis* Dm75, belonging to the same serogroup O78. Visible zones of growth inhibition clearly indicate that the vesicles successfully attached to the living cells and killed them, most probably by delivering the lytic enzymes. Thus, the blebs forcibly prevented the multiplication of the attacked cells on the place they acted. Interestingly, the OMV10 (O78) sample was not as rich in blebs as the OMV8 (O77) sample, and its cross-fusion with the O77 cells was not as productive as in the case of the latter one. However, the strong antagonistic effect of these vesicles on another *P. mirabilis* strain from the O78 serogroup proves its high lytic potential, which requires further studies.

Interestingly, Li et al. [9] showed that *P. vulgaris* OMVs have a killing effect on non-dividing bacterial cells of various Gram-negative and Gram-positive bacteria. However, the attacked microorganisms were mixed with agar without nutrients; therefore, although alive, the cells could not readily repair the cell wall lesions. Our experiments exhibited that the tested OMVs obtained from the *P. mirabilis* O78 strain had an even stronger antagonistic effect, inhibiting the growth of actively dividing cells, which is an additional advantageous feature of these OMVs. However, these properties were not sufficient enough to have an effect on the cells suspended in phosphate saline (PBS) solution. Possibly, the amount of blebs was too low compared to the number of living bacterial cells. Using the same method, Kadurugamuwa et al. [20] could see the impact of *P. aeruginosa* vesicles on the cells of a non-homologous *P. aeruginosa* strain; however, the bactericidal effects on the *E. coli* or *S. aureus* dividing cells in a liquid medium culture were not noted.

Currently, there is a growing interest in outer membrane vesicles, their production, and the functions they perform. The understanding of their mechanisms and their role in the pathogenesis of bacterial diseases, as well as the phenomenon of their incorporation into other bacterial or eukaryotic cells, has a vital importance for the future medical applications of these structures. The comprehension of this process may enable their use in the future for targeted treatment. Therefore, a large amount of research currently concerns the possibility of controlling the production of OMVs by bacteria, and designing these structures with specific features and desired cargo. This is a promising perspective in the treatment of infectious diseases and cancer, and in developing vaccines [30]. The results obtained in our work have enriched the scant knowledge about the phenomenon of OMV production by the bacteria of the genus *Proteus* and has led to the elucidation of certain properties of these structures. The recognition of the interactions of OMVs with bacterial cells is an impulse for the further development of this research topic and the possible applications of *P. mirabilis* OMVs due to their lytic properties.

## 4. Materials and Methods

### 4.1. Bacterial Strains

*P. mirabilis* clinical strains representing the serogroups prevalent in patients from central Poland, including O77 (3Bm) and O78 (1Bm, Dm75), O20 (Sr144), and O11 (Sm98), belong to the collection of the Department of Biology of Bacteria, University of Lodz, Poland. The strains 1Bm, 3Bm, Dm75, and Sm98 were isolated from urine, and Sr144 was isolated from a wound. Their properties were previously described [12,24,31]. In addition, the reference strains *S. aureus* ATCC 29213 and *E. coli* ATCC 25922 were included in the research. The isolates were cultured on LB or nutrient broth (BTL, Lodz, Poland) and stored at −80 °C in the presence of 25% glycerol as frozen stocks.

### 4.2. Determination of Logarithmic Growth Phase

The late logarithmic phase of growth, according to Klimentova et al. [13], is the period when bacteria produce OMVs most intensively. The study was designed for the *P. mirabilis* 1Bm (O78) and 3Bm (O77) strains cultured in 200 mL of nutrient broth medium (in triplicate) and LB medium with aeration in an Aeroton shaker (155 rpm, 37 °C; Infors AG, Bottmingen, Switzerland). Every 2 h, the number of bacteria was estimated. An amount of 100 µL of the diluted culture was spread in duplicate on nutrient agar plates with 2.2% agar, which prevented *Proteus* swarming growth, and, after 24 h of incubation, the CFUs/mL were counted.

### 4.3. OMV Isolation

The OMVs studied in this research were obtained as five independent preparations, as indicated in Table 1. The isolation of the OMVs was performed according to Kulp and Kuehn [32], with some modifications. The tested (Section 4.1) *P. mirabilis* 1Bm or 3Bm liquid cultures were inoculated at a ratio of 1:100 (*v/v*) to 200 mL of LB medium and incubated with aeration in an Aeroton shaker (155 rpm, 37 °C; Infors AG, Bottmingen, Switzerland) for 19 h to the end of the logarithmic growth phase. Subsequently, the bacterial cultures were centrifuged (5285× *g*, 10 °C, 50 min; Sigma-Aldrich, St. Louis, MO, USA) and the supernatants were filtered through a sterile nitrocellulose filter with a pore size of 0.2 µm (Sartorious, Gottingen, Germany) to remove the residual bacterial cells or their fragments. An amount of 185 mL of the obtained filtrates was ultracentrifuged (150,000× *g*, 4 °C, 3 h; Beckman L-80 Optimum Class R, Brea, CA, USA) and the pellets of the OMVs were suspended in 2 mL of a sterile PBS solution. Bacterial biomasses which remained after cultivation were autoclaved, freeze-dried, and weighed in order to calculate the efficiency of the OMV production, which was calculated per 100 mg of dry bacterial cells based on the following formula: the amount of LPSs building the OMVs/dry bacterial biomass × 100. Visualisation in TEM (Section 4.5.1), ELISA (Section 4.5.2), and Western blotting (Section 4.5.3) assays were employed to confirm the presence of OMVs in the prepared samples. The size of the *P. mirabilis* cells and O77 blebs (OMV3), as well as zeta potential of the vesicles, were determined using the Zetasizer Ultra Red (Malvern Panalytical Ltd., Malvern, UK).

### 4.4. Assay of OMV Fusion to Bacterial Cells

In order to examine whether the obtained OMVs could be integrated with the outer membrane and connected with the recipient cells, a fusion test was performed based on the method described by Renelli et al. [33], with some modifications. Twenty-four-hour cultures of *P. mirabilis* 1Bm or 3Bm strains were diluted in PBS to the OD_550_ = 0.33 MF (DEN 1-B McFarland densitometer, SIA Biosan, Riga, Latvia) (1 × 10^8^ CFUs/mL). The cell suspensions were mixed in the ratio 10:1 with a suspension of the previously obtained non-homologous OMVs. *S. aureus* and *E. coli* reference strains were used as controls. All of the samples were incubated at 37 °C for 30 min. In the next step, to remove the unbound vesicles, the samples were centrifuged (12,000 rpm, 15 min) and the cell sediments were washed in 800 µL of PBS and centrifuged under the same conditions. Cell washing was repeated 3 times, and the obtained precipitates were suspended in 100 µL of PBS. To confirm the integration of the OMVs with the recipient bacteria, ELISA and Western blotting techniques were performed.

### 4.5. Confirmation of OMV Isolation and Fusion Phenomenon

Due to the small volume (2 mL) of the OMV samples, the ELISA experiments confirming the presence and amounts of blebs in the obtained OMV samples were made in triplicate for the OMV samples and five times for the LPSs (controls), while the other tests were conducted once for each OMV sample.

#### 4.5.1. Visualisation of OMVs in TEM

For the TEM, the suspensions of bacterial cells or OMVs (10 µL) were placed on a 200-mesh formvar carbon-coated copper grid (Ted Pella Inc., Redding, CA, USA) for 5 min. The excess of the sample was blotted away with filter paper and the grid was rinsed with distilled water. Then, the sample was negatively stained with 2% uranyl acetate for 30 s. The excess of the stain was wicked away with filter paper and the gird was air-dried. The analyses were performed using the JEM-1010 (Jeol, Tokyo, Japan) TEM at an accelerating voltage of 80 kV. Images were recorded on 6.5 × 9 cm electron microscopy imaging films (Maco Photo Products, Stapelfeld, Germany); then, after their development, the films were scanned using a Perfection V700 Photo scanner (Epson, Suwa, Japan).

#### 4.5.2. ELISA

In the ELISA method, the wells in the first column of a 96-well polystyrene flat-bottom Nunc-Immuno plate were coated with 50 µL of the tested antigens in PBS, which were the OMVs produced by the *P. mirabilis* O77 or O78 strains or the bacterial recipient cells after the fusion assay. The biomass (5 µg in 50 µL of PBS per well) of the *P. mirabilis* strains producing the tested OMVs, the *S. aureus* or *E. coli* reference strains, as well as the extracted lipopolysaccharides (50 ng in 50 µL PBS per well) from the respective *P. mirabilis* strains were employed as control antigens. The antigens were serially diluted (q = 2) in PBS and incubated for 24 h at 4 °C. After that, the uncoated area in the wells was blocked (1 h, 37 °C) with casein buffer at a pH = 7.2 (2.5% casein, 1.5 mM KH_2_PO_4_, 240 mM NaOH, and 8 mM Na_2_HPO_4_) and then washed with distilled water and PBS. *P. mirabilis* O77- or O78-specific sera diluted at a ratio of 1:1000 in casein buffer (50 µL) were added to the wells, incubated (1 h, 37 °C), and then washed three times in PBS. Subsequently, rabbit-IgG-specific peroxidase-conjugated goat antibodies (Jackson ImmunoResearch, West Grove, PA, USA) diluted at a ratio of 1:5000 in casein buffer were added to the plate, incubated (1 h, 37 °C), and washed three times with PBS and once with a substrate buffer at a pH = 4.5 (100 mM C_6_H_5_Na_2_O_7_·2H_2_O). In the last stage, the plate was incubated (30 min, 37 °C) with a solution of 2,2′-Azino-bis(3-ethylbenzothiazoline-6-sulfonic acid) diammonium salt (ABTS, Sigma-Aldrich, St. Louis, MO, USA) in the substrate buffer (5 µg in 1 µL with the addition of 0.1% H_2_O_2_), which was a substrate to peroxidase. After that, the reaction was stopped by adding 50 µL of 222 mM C_2_H_2_O_4_ per well. The absorbance was measured using a microplate reader, the Multiskan Go (Thermo Fisher Scientific, Waltham, MA, USA, Vantaa, Finland), at a wavelength of 405 nm. An extinction ≥ 0.2 was considered as indicating a positive reaction.

#### 4.5.3. Western Blotting

An amount of 20 µL of each tested (OMVs, bacterial cells after fusion with OMVs) or control (bacterial cells, LPSs) antigen in PBS was added to 20 µL of sample buffer (2% SDS and 50 mM Tris/HCl (pH of 6.8), 25% glycerol, and 0.1% bromophenol blue) and disintegrated for 10 min at 100 °C. Subsequently, 5 µL of Proteinase K (A&A Biotechnology, Gdansk, Poland) was added to the samples and then incubated for 1 h at 56 °C to digest the cellular proteins. The samples were separated during sodium dodecyl sulphate polyacrylamide gel electrophoresis (SDS PAGE) at a voltage of 200 V and then transferred onto a nitrocellulose membrane. Electrotransfer was carried out for 1 h at 4 °C at a voltage of 100 V. After that, the membrane was blocked in 10% skimmed milk in a dot-blot buffer at a pH of 7.4 (50 mM Tris/HCl and 200 mM NaCl) and subsequently incubated with an appropriate *P. mirabilis* O77- or O78-specific serum, diluted at a ratio of 1:100 in the same blocking buffer, for 1.5 h. After washing in dot-blot buffer, the membrane was incubated overnight with alkaline phosphatase-conjugated goat anti-(rabbit IgG) antibody (Jackson ImmunoResearch, West Grove, PA, USA) diluted at a ratio of 1:10,000 in blocking buffer. After incubation, 5-bromo-4-chloro-3-indoylphosphate p-toluidine and p-nitroblue tetrazolium chloride (Bio-Rad, Warsaw, Poland) were added as the substrate for alkaline phosphatase to visualise the separated lipopolysaccharides.

### 4.6. Bacteriolytic Activity of OMVs

The lytic activity of the OMVs was determined according to Kadurugamuwa and Beveridge [10] against the *P. mirabilis* O77, O78, O20, and O11 strains, as well as the *S. aureus* and *E. coli* cells, which were washed with PBS to remove the culture medium. The non-homologous samples of the membrane vesicles were added to a suspension (1.5 × 10^8^, 0.5 MF) of the bacterial cells in the ratio of 1:10 (*v/v*). The suspensions of the respective strains without the OMVs were used as a control. The experiments were performed from 0 to 5 h at 37 °C. At 0, 1, 3, and 5 h of incubation, the OD_550_ of the samples was measured using a DEN 1-B McFarland densitometer (SIA Biosan, Riga, Latvia), and the 100 µL aliquots from a dilution of 10^−5^ were spread in duplicate on nutrient agar plates (2.2% agar, preventing swarming growth). After 24 h of incubation, the colonies were counted and the results were presented as CFUs/mL. Due to the huge volume of OMV samples necessary to perform this assay, it could be performed in one replication.

In order to examine the OMV lytic properties, a spot assay was performed in triplicate according to the Afoshin et al. [34] method, with some modifications. The tested bacterial cultures (1 × 10^8^ CFUs/mL) were spread on the surface of the LB agar 1.5% plates. Then, 10 µL each of the OMV samples were added as spots. They were then incubated overnight at 37 °C. The results of the OMV lytic action were observed as the bacterial growth inhibition in the spot areas (as shown in Figure 6).

## Figures and Tables

**Table 1:** The efficiency of the OMV production by the tested *P. mirabilis* strains.

OMV Sample (The Producing Strain; Serogroup)	Dry Bacterial Biomass [mg]	Amount of LPSs in the OMV Sample [ng/2 mL]	Efficiency (OMV LPSs per 100 mg of Dry Biomass [ng])
OMV3 (*P. mirabilis* 3B-m O77)	199.56	2000	1002.2
OMV8 (*P. mirabilis* 3B-m O77)	199.18	12,000	6024.7
OMV9 (*P. mirabilis* 3B-m O77)	167.85	2000	1191.5
OMV7 (*P. mirabilis* 1B-m O78)	181.04	520	287.2
OMV10 (*P. mirabilis* 1B-m O78)	298.23	2000	670.62

## Data Availability

The data that support the findings of this study are available from the corresponding author on reasonable request.

## Supplementary Materials

The following supporting information can be downloaded at: https://www.mdpi.com/article/10.3390/molecules29204836/s1, Table S1: The viability (CFUs/mL) in time of the studied strains in nutrient broth medium.; Table S2: Reactions in ELISA (absorbance mean values ± SD) of OMV and LPS samples (antigens). The data (mean values) in the form of charts are presented in Figure 3A,B.
