# Supplementary material for: Outer Membrane Vesicles Formed by Clinical Proteus mirabilis Strains May Be Incorporated into the Outer Membrane of Other P. mirabilis Cells and Demonstrate Lytic Properties
Source: Molecules. 2024 Oct 12;29(20):4836. doi: 10.3390/molecules29204836 (PMC11509992; doi:10.3390/molecules29204836)
Supplement: Supplementary file 1 [file molecules-29-04836-s001.zip › molecules-3199174-supplementary.pdf]

Supplementary file

Outer membrane vesicles formed by clinical *Proteus mirabilis* strains may be incorporated into the outer membrane of other *P. mirabilis* cells and demonstrate lytic properties

Table S1. The viability (CFU/mL) in time of the studied strains in nutrient broth medium.

Bacterial culture [hour]	Mean of CFU/mL (×10^9) ± SD	
	P. mirabilis O77	*P. mirabilis* O78
4	0,125 ± 0,0636	0,055 ± 0,035
6	1,62 ± 1,3	0,455 ± 0,092
8	5,24 ± 1,3	2,74 ± 0,54
10	7,2 ± 1,36	5,92 ± 0,79
12	10,8 ± 3,32	6,02 ± 0,88
14	9,57 ± 1,95	7,49 ± 1,46
16	9,15 ± 2,69	9,93 ± 3,38
18	9,42 ± 0,984	9,92 ± 1,07
20	11,3 ± 1,06	11,6 ± 2,54
22	9,91 ± 3,76	11,4 ± 2,38
24	9,36 ± 1,91	10,3 ± 1,64
26	9,06 ± 2,74	8,95 ± 0,72
28	9,93 ± 1,33	8,99 ± 2,42
30	1,06 ± 0,0283	9,29 ± 1,15
32	9,1 ± 1,56	9,46 ± 1,61

Table S2. Reactions in ELISA (absorbance mean values ± SD) of OMV and LPS samples (antigens). The data (mean values) in the form of charts are presented on the Figure 3 A,B.

Antigen dilution	OMV3 (O77)	OMV7 (O78)	OMV8 (O77)	OMV9 (O77)	OMV10 (O78)	LPS O77	LPS O78
1	1.446 ± 0.274	0.485 ± 0.212	3.105 ± 0.205	1.563 ± 1.015	1.283 ± 0.548	2.214 ± 0.911	2.000 ± 0.585
1:2	1.443 ± 0.143	0.491 ± 0.062	2.880 ± 0.424	1.338 ± 0.767	0.941 ± 0.466	2.228 ± 0.845	1.97 ± 0.478
1:4	1.603 ± 0.162	0.452 ± 0.041	2.875 ± 0.459	1.237 ± 0.514	0.793 ± 0.449	2.076 ± 0.881	1.588 ± 0.301
1:8	1.567 ± 0.247	0.322 ± 0.051	2.750 ± 0.537	1.209 ± 0.398	0.684 ± 0.410	2.044 ± 0.864	1.228 ± 0.110
1:16	1.393 ± 0.155	0.228 ± 0.017	2.655 ± 0.728	1.300 ± 0.269	0.559 ± 0.261	1.866 ± 0.681	1.082 ± 0.209
1:32	1.113 ± 0.110	0.155 ± 0.011	2.580 ± 0.750	1.040 ± 0.284	0.545 ± 0.140	1.621 ± 0.581	0.616 ± 0.133
1:64	0.953 ± 0.180		2.420 ± 1.117	0.909 ± 0.284	0.458 ± 0.141	1.126 ± 0.367	0.367 ± 0.055
1:128	0.844 ± 0.137		2.245 ± 1.210	0.577 ± 0.162	0.298 ± 0.132	0.763 ± 0.336	0.232 ± 0.055
1:256	0.530 ± 0.073		2.025 ± 1.393	0.544 ± 0.023	0.280 ± 0.000	0.567 ± 0.172	0.177 ± 0.053
1:512	0.356 ± 0.067		1.738 ± 1.106	0.459 ± 0.025		0.386 ± 0.236	
1:1024	0.196 ± 0.064		1.567 ± 1.249	0.304 ± 0.019		0.263 ± 0.154	
